# A study of correlations between cephalometric measurements in Koreans with normal occlusion by network analysis

**DOI:** 10.1038/s41598-024-60410-1

**Published:** 2024-04-26

**Authors:** Seorin Jeong, Sehyun Kim, Sung-Hoon Lim, Sun-Kyoung Yu

**Affiliations:** 1 Department of Orthodontics, College of Dentistry, Chosun University, 7 Chosundaegil, Dong-Gu, Gwangju, South Korea; 2 Department of Oral Anatomy, College of Dentistry, Chosun University, 7 Chosundaegil, Dong-Gu, Gwangju, South Korea

**Keywords:** Oral anatomy, Malocclusion, Craniofacial orthodontics, Malocclusion

## Abstract

Analyzing the correlation between cephalometric measurements is important for improving our understanding of the anatomy in the oral and maxillofacial region. To minimize bias resulting from the design of the input data and to establish a reference for malocclusion research, the aims of this study were to construct the input set by integrating nine cephalometric analyses and to study the correlation structure of cephalometric variables in Korean adults with normal occlusion. To analyze the complex correlation structure among 65 cephalometric variables, which were based on nine classical cephalometric analyses, network analysis was applied to data obtained from 735 adults (368 males, 367 females) aged 18–25 years with normal occlusion. The structure was better revealed through weighted network analysis and minimum spanning tree. Network analysis revealed cephalometric variable clusters and the inter- and intra-correlation structure. Some metrics were divided based on their geometric interpretation rather than their clinical significance. It was confirmed that various classical cephalometric analyses primarily focus on investigating nine anatomical features. Investigating the correlation between cephalometric variables through network analysis can significantly enhance our understanding of the anatomical characteristics in the oral and maxillofacial region, which is a crucial step in studying malocclusion using artificial intelligence.

## Introduction

Recently, attempts to apply data-driven techniques including artificial intelligence, to the field of orthodontics have been active^1–3^. The number of studies involving various orthodontic applications of artificial intelligence and machine learning has grown exponentially, with the most commonly studied areas being diagnosis and treatment planning, automated anatomical landmark detection and/or analysis, and growth and development assessment^4^. In particular, to conduct data-driven research in the diagnosis and treatment plan, it is crucial to examine how malocclusion is understood as data.

Malocclusion is a commonly observed dentofacial abnormality with various classifications for its phenotype and underlying causes. However, it is essentially a disharmonious relationship between different elements of the oral and maxillofacial region. Cephalometric analysis using lateral cephalometric radiographs is a widely used approach for diagnosing malocclusion and evaluating orthodontic treatment. Most cephalometric measurement sets used in clinical or research applications are recombination of various classical cephalometric analyses^5–21^. For treatment and research purposes, clinicians and researchers typically utilize various sets which are composed of 10–20 cephalometric measurements and customized according to user preferences. However, this practice can lead to biased results, which make it difficult to directly compare different studies. Therefore, it is essential to discover a standardized input set of cephalometric measurements that can comprehensively depict the oral and maxillofacial regions. To reach this objective, it is crucial to comprehend the correlation structure among elements.

The analysis of networks is a useful method for examining complex systems involving numerous connections among different elements. Its usefulness has been established in diverse fields, ranging from social networks to biological systems^22–26^. Furthermore, in dentistry, network analysis has been validated as a tool to analyze and visualize the structure of correlations between anatomical features of the oral and maxillofacial region, a complex system in which various elements interact^4,27–35^. As in prior research, the characteristics of patients with malocclusion, specifically cephalometric measurements, were represented as nodes in the network, and the weights of the network edges reflected the correlations between a pair of measurements^27–32,35^. Moreover, these previous studies employed rule-of-thumb methods for determining cutoff points to be applied to link weights during network analysis. However, to achieve better results than previous studies, it is necessary to comparatively analyze the network structure and consider the statistical distribution of edge weights to determine the appropriate cutoff.

Previous studies have studied correlation network structures of malocclusion patients from 4 to 20 years of age, spanning growth through primary dentition, mixed dentition, and permanent dentition^27–32,35^. These studies revealed a robust correlation structure in network during the growth process and the importance of the kernel structure in the treatment of malocclusions^28,29^. Differences in the network morphology were observed among patient groups^27,29–31^. The results of the network analysis were very helpful in predicting the treatment prognosis of patients in the growth phase^31,32,35^.

However, further research is required to expand and enhance the studies in various aspects. Initially, there is a need for research on the permanent dentition, which is when the malocclusion phenotype is completed. Establishing a treatment plan for childhood malocclusion and predicting the prognosis after growth is helpful in interceptive orthodontics. Nevertheless, conducting research on adults is crucial in treating malocclusions that are finalized after growth, and such research can have a significant clinical impact.

In addition, to conduct research on different malocclusions, it is necessary to conduct research on normal occlusion as a reference. The majority of previous studies have focused on class III malocclusion^28–31,35^, while one study confirmed differences among networks of class I, II, and III malocclusions^27^. However, normal occlusion has not been studied in this way. Studying normal occlusion as a reference and the ultimate treatment goal is essential to thoroughly compare and analyze different malocclusions.

Therefore, the aims of this study were to understand and analyze the correlation structure between different anatomical elements in the oromaxillofacial region in Korean adults with normal occlusion using weighted network analysis methodology and the minimum spanning tree (MST) algorithm. This algorithm identifies the core structure of the network by minimizing the sum of path costs while selecting a subset of the network structure where all elements are connected without forming loops.

## Materials and methods

### Participants

This study received approval from the Institutional Review Board of Chosun University Dental Hospital under the reference number CUDHIRB 1901 009 R01. Due to the retrospective framework of the study utilizing anonymized data, the requirement for informed consent was exempted by the Institutional Review Board of Chosun University Dental Hospital. All protocols were executed in adherence to pertinent guidelines and regulations.

We used data from 753 patients with normal occlusion, which was originally created for “report on cephalometric measurement of Korean adult with normal occlusion” that was published by the malocclusion white paper publication committee of the Korean association of orthodontics^36^. This data is the result of a collaborative study conducted in the past by 10 dental schools in South Korea that operate independent dental clinics, and is the only large-scale survey of a Korean sample of normal occlusion to date. The present study was conducted using data from this study.

The present study defined individuals with normal occlusion as those meeting the following criteria:

1. Class I molars and canines relationship,
2. Presence of all teeth except wisdom teeth,
3. Prosthetic treatment performed on less than 1 side,
4. Interdental spacing less than 1 mm,
5. Crowding less than 3 mm,
6. Horizontal or vertical overlay between 2 and 4 mm, and,
7. Midline displacement less than 1 mm.

Analysis was conducted on 735 individuals between the ages of 18 and 25, comprising 368 males and 367 females, with no missing or outlier measurement data.

### Cephalometric variables

This study developed a list of variables based on nine well-known cephalometric analysis methods, to ensure they are universal. We removed repeated variables (e.g. Facial angle (Downs) & Facial depth (Ricketts)) in lateral cephalometric measurements used in Downs, Tweed, Steiner, Coben, Jarabak, Jacobson (‘Wits’ appraisal), Ricketts, McNamara, and Kim analysis methods^6–21^. Consequently, we compiled a list of 96 variables. We further excluded variables that had a geometric correlation of ‘ + 1’ (e.g. Sum & Sn-GoGn) and those that could not be obtained from the data or had significant data loss. This study utilized a final list comprising 65 variables, which are elaborated on in Table 1. In addition, Table S1 offers a comprehensive description of each variable.

**Table 1:** The variables list of cephalometric measurements performed in this study.

No.	Cephalometric variables	Unit	No.	Cephalometric variables	Unit	No.	Cephalometric variables	Unit
[1]	Incisor overjet	mm	[23]	ANB	deg	[45]	Body to anterior cranial base ratio	ratio
[2]	Incisor overbite	mm	[24]	U1 to NA	mm	[46]	Facial depth	mm
[3]	Interincisal angle	deg	[25]	U1 to NA Angle	deg	[47]	Facial length on Y-axis	mm
[4]	Convexity of point A	mm	[26]	L1 to NB	mm	[48]	Posterior facial height	mm
[5]	Lower facial height	deg	[27]	L1 to NB Angle	deg	[49]	Anterior facial height	mm
[6]	Upper molar to PTV	mm	[28]	Pog to NB	mm	[50]	Posterior to anterior facial height ratio	ratio
[7]	L1 to A-Pog	mm	[29]	Pog & L1 to NB (diff.)	mm	[51]	Occlusal plane to GoGn	deg
[8]	U1 to A-Pog	mm	[30]	Occlusal Plane to SN	deg	[52]	U1 to SN angle	deg
[9]	L1 inclination	deg	[31]	Wits Appraisal	mm	[53]	U1 to facial plane	mm
[10]	Lower lip to E-plane	mm	[32]	IMPA	deg	[54]	L1 to facial plane	mm
[11]	Facial angle	deg	[33]	FMIA	deg	[55]	Lower anterior facial height	mm
[12]	Facial axis	deg	[34]	Saddle angle	deg	[56]	Upper Lip to E-Plane	mm
[13]	FMA	deg	[35]	Articular angle	deg	[57]	Nasion perpendicular to point A	mm
[14]	Palatal plane angle	deg	[36]	Gonion angle	deg	[58]	Pog-N perpendicular	mm
[15]	Mandibular Arc	deg	[37]	Sum	deg	[59]	U1 to A vertical	mm
[16]	Facial convexity	deg	[38]	Anterior cranial base	mm	[60]	ODI	deg
[17]	A-B plane angle	deg	[39]	Posterior cranial base	mm	[61]	A-B to mandibular plane	deg
[18]	Y axis	deg	[40]	Upper gonial angle	deg	[62]	APDI	deg
[19]	Occlusal plane angle	deg	[41]	Lower gonial angle	deg	[63]	Combination factor	deg
[20]	L1 to Occlusal plane angle	deg	[42]	Ramus height	mm	[64]	Extraction Index	complex
[21]	SNA	deg	[43]	Posterior cranial base to ramus ratio	ratio	[65]	U1 to FH plane	deg
[22]	SNB	deg	[44]	Mandibular body length	mm			

### Correlation analysis and network analysis

The correlation structure of 65 measurement variables was analyzed using Pearson’s correlation coefficient and network analysis. As in previous studies, we defined each measurement variable related to anatomical characteristics of the oral and maxillofacial region as a node within the network^27–32,35^. The sign of each measured variable is defined individually in each cephalometric analysis, and it is significant when analyzing the correlation between two variables, but there is no single standard for definition of signs of all variables. Thus, when evaluating the correlation structure between multiple anatomical features, it is worthwhile to use the absolute values of the correlation coefficients. The absolute values of correlation coefficients were used as the weights of the connections between all pairs of nodes, referred to as edge weights. Instead of considering all correlations between variables in a weighted network, a threshold was set to include only cases with weights above this cutoff, similar to the prior studies that excluded correlations with absolute values below 0.6^27–32,35^. This allows for a more intuitive visualization and analysis of intricate networks while preserving as much information as possible.

To visualize complex network structures, not only weighted networks but also minimum spanning tree was used^37^. Although this method loses considerable information, it offers a straightforward way of visualizing the overall structure of the network. Similar to other studies involving correlation networks for minimum spanning trees, we defined the distance between nodes in the network as the difference of 1 and a correlation coefficient’s absolute value^38,39^. The MST structure was obtained using the Kruskal algorithm, which minimizes the sum of all residual paths while removing all loops^40^. This approach allows for identifying and analyzing the most important structures of complex networks. As a result, this method makes it easier to understand the intercorrelation structure among clusters of cephalometric features.

## Result

**Figure 1:**
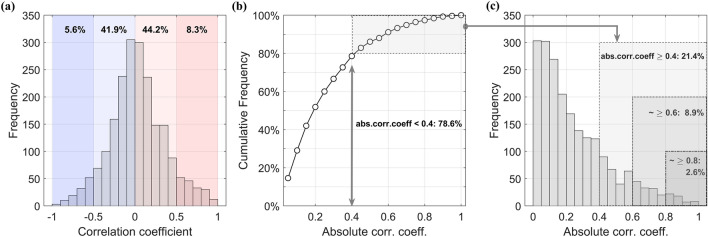
(**a**) Histogram of Pearson’s correlation coefficients between 65 cephalometric measurements. (**b**) Cumulative probability distribution function (CPDF) of absolute values of correlation coefficients. (**c**) Histogram of absolute values of correlation coefficients.

Figure 1 illustrates the statistical distribution of Pearson’s correlation coefficients in order to set appropriate cutoffs before analyzing the structure of the 2,080 correlations between the 65 cephalometric variables. Figure 1a shows a distribution of the overall correlation coefficient that is approximately bell-shaped, though with a slight positive skew. As previously stated, the distribution of the absolute value of the correlation coefficient is more crucial, and Fig. 1b displays this distribution. According to the cumulative probability distribution function, values below 0.4, which are relatively insignificant, account for 78.6% of the entire distribution. A minimum cutoff below this level results in a high percentage of correlations considered, which is not suitable for meaningful interpretation.

Hence, the minimum cut-off value has been set at 0.4. Figure 1c shows the histogram of absolute correlation coefficients, showing that selecting coefficients of 0.4 or more, 0.6 or more and 0.8 or more includes 21.4%, 8.9% and 2.6% of total correlations, respectively. Subsequently, the study proceeds with a network analysis using these three cut-off values and evaluates their appropriateness. As a result, this procedure yields improved outcomes compared to previous studies that relied on empirical cut-off values.

**Figure 2:**
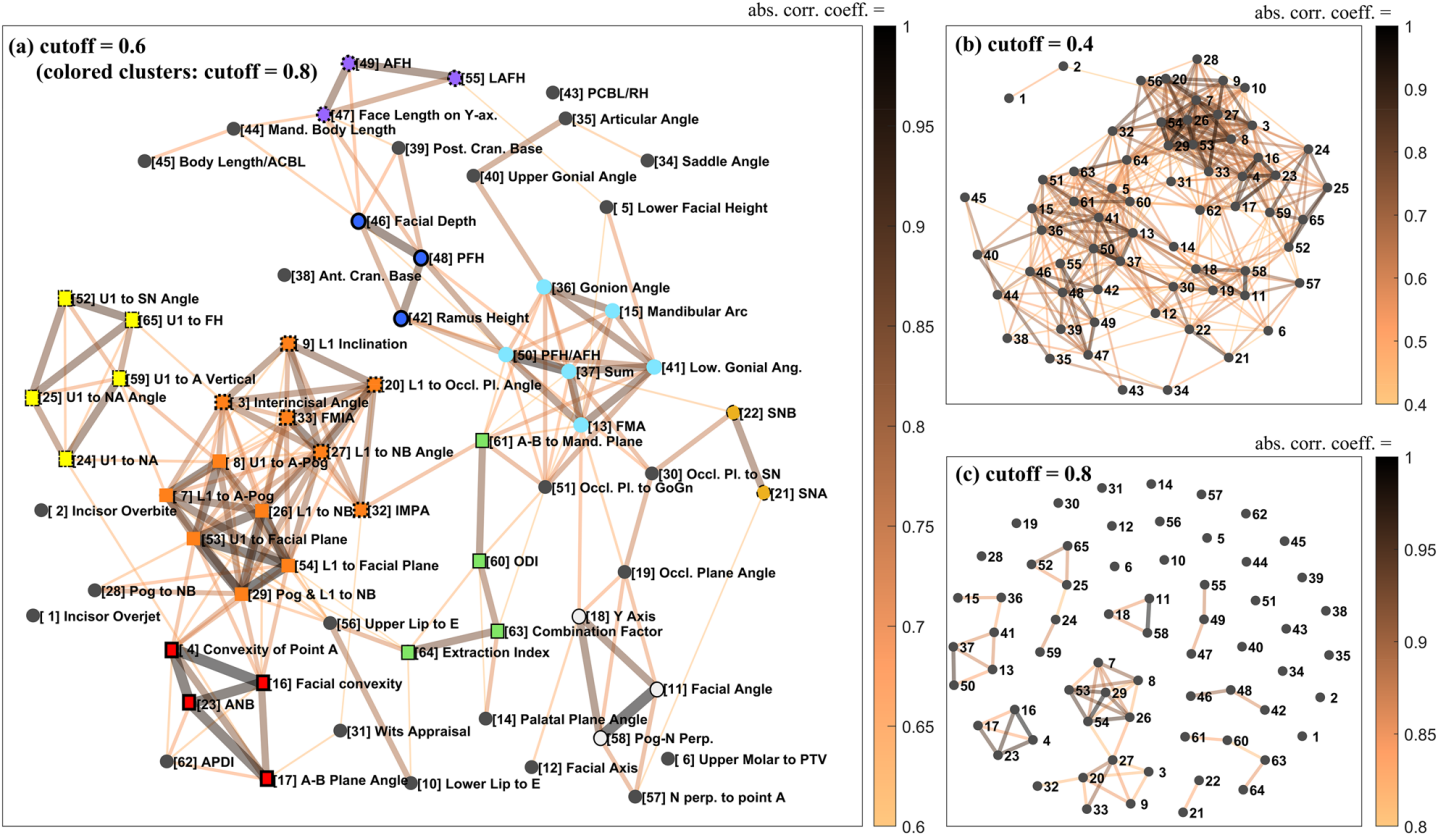
Weighted correlation networks of the 65 cephalometric variables. (**a**) applied a cutoff of 0.60 and the correlation network with correlation coefficients greater than 0.60 in absolute value was connected by a line (colored clusters show isolated clusters when cutoff = 0.80 is applied). (**b**) cutoff = 0.40 and (**c**) cutoff = 0.80.

Figure 2 displays the weighted network of the correlation structure of the cephalometric variables applied with cutoffs of 0.4, 0.6 and 0.8, where stronger correlations are indicated by darker and thicker lines. Figure 2a shows that, even when we exclude information from the colored clusters, applying a cutoff of 0.6 results in the establishment of multiple clusters. This confirms the existence of an inter- and intra-correlation structure between anatomical features that is beneficial for understanding the oral and maxillofacial region. Figure 2b shows that using the network with a cutoff of 0.4 contains more information, but the structure of the network is too complex to extract any meaningful information. Moreover, applying a 0.8 cutoff in Fig. 2c is more intuitive, revealing a highly correlated cluster structure. However, this approach has drawbacks, such as removing a significant amount of information, including correlations between groups.

Considering the highly correlated groups obtained at cutoff = 0.8, the information can be represented as “colored clusters” in the network at cutoff = 0.6, as shown in Fig. 2a. This representation improves the intuitiveness of the clusters while still including sufficient inter- and intra-correlation information. In the graph, groups of the same colored nodes, except black, represent isolated clusters revealed at cutoff = 0.8. The largest cluster at the bottom of Fig. 2c is divided into an upper and lower part based on node 27, and while these parts are highly correlated individually, the two parts are weakly correlated with each other. In Fig. 2a, the two subsets marked by the same orange square nodes are distinguished by a difference in outline. As noted in Table 2, the groups of variables shown in Fig. 2a mostly correspond to the major anatomical features of the oral and maxillofacial region.

**Table 2:** Anatomical characteristics in the oral and maxillofacial regions associated with each variable group.

Group	Related anatomical features of the oral and maxillofacial region
A-1	The anteroposterior positions of the maxillary and mandibular incisors relative to the facial anterior part
A-2	The inclination of the mandibular incisors
B	The inclination of the maxillary incisors
C	The anteroposterior relationship between the maxilla and the mandible / Facial convexity
D	Variables related to overbite depth index (ODI)
E	The divergent and the convergent facial types
F	The vertical length of facial posterior part
G	The vertical length of facial anterior part
H	The anteroposterior positions of the maxilla and mandible in relation to the cranial base
I	Mandibular protrusion and the growth direction of the mandible

**Figure 3:**
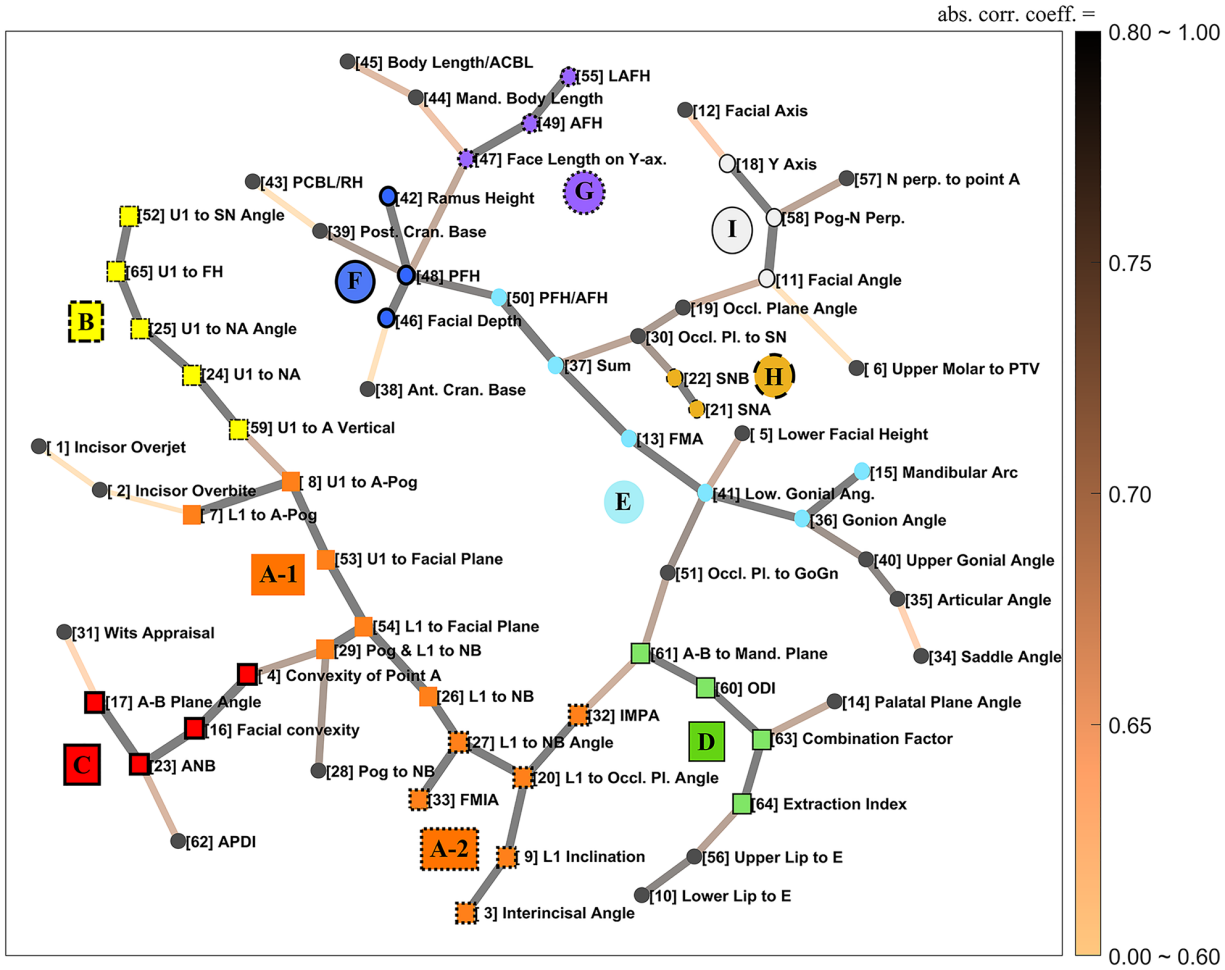
A visualization of the simplified correlation structure of cephalometric variables using minimum spanning tree (MST) and isolated cluster information of Fig. 2(c).

We used the MST algorithm to derive a minimal skeletal structure of the network. This structure shows the connectivity between variable clusters in an intuitive way. The use of this algorithm eliminates the need for any arbitrary cutoff that may be applied in a weighted network. The skeletal structure of the entire network, obtained using MST, is presented in Fig. 3. Highly clustered groups were identified and labeled as nodes of the same color. The labeling was done based on the strongly clustered group information presented in Fig. 2c, as shown in Fig. 2a. The diagram illustrates the structure of the connections between the 10 most highly correlated groups, along with the most significant connections between individual variables. In this case, one can identify the two directly connected groups and the pair of variables that exhibit the highest connectivity intuitively, which is more obvious than in a weighted network.

## Discussion

This study utilized a wide dataset and employed a data-driven analysis process to produce objective results. The findings on normal occlusion in Koreans hold significant value for future studies on malocclusion in this ethnic group, as oral and maxillofacial characteristics and malocclusion patterns can vary among different ethnicities^41^. In addition, the results revealed several significant issues.

Firstly, this study’s analyses cover a more extensive range than previous studies, but they exhibit consistency in overlapping areas. Secondly, interpreting the geometric aspects of variables enables us to distinguish subtle differences between variables that are known to represent the same feature in clinical practice. Thirdly, there were instances where variables known to represent different traits were linked eventually to the same anatomical feature. Finally, through the analysis of the correlation structure of the cephalometric measurement set, we identified nine key characteristics of the oral and maxillofacial region. We also identified clusters of variables that exhibit these characteristics and investigated the inter-correlations between the groups.

In order to confirm the validity of the findings in this study, a comparison with previous studies revealed a significant level of consistency. Most previous researches have focused primarily on class III malocclusions in young patients, which makes it challenging to compare with adult malocclusions analyzed in this study. The only exception was a study by Scala et al. that included a section on adult females with Class III malocclusions^28^, and we were able to compare their results with ours. Out of the 17 variables that were measured in the prior study, 14 either were included or have a significant similarity to the 65 variables examined in this study. Therefore, we constructed an unweighted network (with a cutoff at 0.6) to assess the 91 correlations between those 14 variables. As a result, it was confirmed that the structure was completely identical, except for one case (Wits—ANB). These findings are deemed acceptable because the variables associated with maxillofacial anteroposterior discrepancy may exhibit different patterns within the class III malocclusion and normal occlusion groups. So, there is a high degree of consistency between the two studies. This comparison covers only 4.38% of the variable pairs examined in this study, but this is because the current study is based on a broader data set than previous studies.

ANB and Wits appraisal, previously mentioned as exceptions, are commonly used as clinical indicators representing the degree of maxillomandibular anterior–posterior positional discrepancy. A comparison was made between the correlation coefficients of four indices: [17] A-B plane angle, [23] ANB, [31] Wits appraisal, and [62] APDI ([No.] refers to the corresponding cephalometric variable in Table 1.). All four were clinically accepted as representative of the same anatomical feature. It was found that the first two variables had a strong correlation of 0.95. However, Wits-ANB, which also falls into this category, had a relatively lower correlation of 0.56. To further investigate these aspects, the correlation structure of these four variables and other closely related variables with strong correlations was analyzed in detail.

Despite the fact that the four variables are clinically known to represent the same anatomical feature, two of them exhibit a strong associate structure with the facial convexity, one has moderate correlations, and one does not belong to the cluster. Although the four nodes mentioned earlier exhibit correlated structure in the lower left of Fig. 3, they do not form a single cluster. Group C, represented by the red square nodes and including two of the four nodes, is a cluster exhibiting strong correlation (> 0.8) between two maxillofacial anterior–posterior disparity variables ([23] ANB and [17] A–B plane angle) and two anterior facial convexity variables ([4] convexity of point A and [16] facial convexity). In this case, a group of nodes in which each node is strongly connected to every other node is known as a clique structure^28^. In addition, Fig. 2a shows that [31] Wits appraisal and [62] APDI, which are not included in Group C, show differences in their connectivity. APDI is not strongly connected to group C, but forms a clique structure with moderate correlations (0.63–0.69) with all nodes in this group. On the other hand, [31] Wits appraisal showed weak correlations (0.47, 0.47, 0.56 and 0.62) with the corresponding group, leading to its exclusion from the cluster.

**Figure 4:**
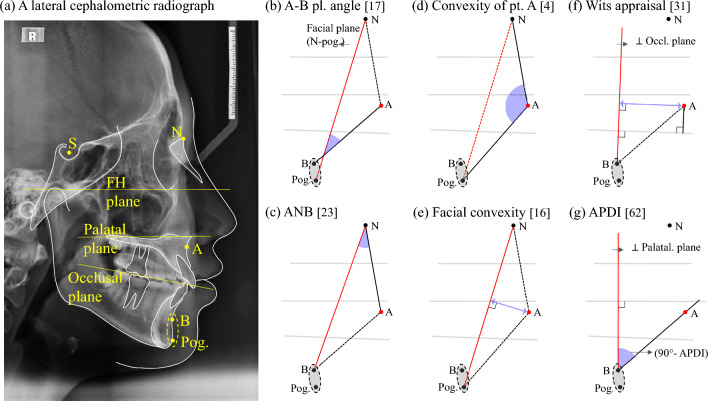
Geometric analysis of indices evaluating the anterior–posterior positional relationship of the maxilla and mandible and neighboring variables on the network. (**a**) Major landmarks & reference planes on a lateral cephalometric radiograph, (**b**) A–B plane angle, (**c**) ANB, (**d**) Convexity of point A, (**e**) Facial convexity, (**f**) Wits appraisal, and (**g**) 90° – APDI.

As can be seen in Fig. 4a, we considers point B and the pogonion to be in approximately the same position from an anterior–posterior perspective, and a geometric analysis was conducted for the six indices. In all the cases of Fig. 4b–e, the anteroposterior position of the point A is evaluated in relation to the vertical plane of the anterior surface. However, it can be divided into three cases according to the reference planes. Figure 4b–e shows that [17] A–B plane angle, [23] ANB, [4] Convexity of point A and [16] Facial convexity all use the facial plane (N-pog.) as the vertical reference line. Furthermore, a geometric relationship of the [31] Wits appraisal depicted in Fig. 4f, shows that it estimates the anteroposterior position of the point A relative to the perpendicular of the occlusal plane passing through point B. On the other hand, the [62] APDI index is defined as the sum of the [11] Facial angle, [17] A–B plane angle, and [14] palatal plane angle^21^. Alternatively, the angle of the A–B plane in relation to the palatal plane can replace it based on the geometric relationship^42^. Next, this index is converted into a measure of the anteroposterior position of point A to a line perpendicular to the palatal plane passing through point B, as shown in Fig. 4g.

By geometrically transforming and interpreting the variables representing the degree of convexity of the anterior face and the variables representing the anteroposterior positional relationship of the maxilla and mandible, it was found that variables known to represent the same anatomical characteristics in clinical practice are sometimes subdivided and variables representing different characteristics are sometimes grouped together. The difference between the correlation configurations of the [31] wits appraisal and the [62] APDI is also interpreted in this way. The landmarks, N and the palatal plane, which determine the vertical reference planes, are equally located on the skeletal structure, so that the [62] APDI shows a moderate correlation with the variables in group C. However, the occlusal plane, which is the reference of the [31] wits appraisal, is a dental structure, so that it is estimated that it shows a low correlation.

The inter-correlation structure among clusters applying cutoff value of 0.6 in Fig. 2a supports the presence of nine major anatomical characteristics in cephalometric variables. All groups, except for group A-1, exhibit a weak inter-correlation with each other. This indicates that the primary anatomical features represented by groups A-2, B, C, E, F, G, H, and I, whose meanings are described in Table 2, are independent features. The anteroposterior positions of the maxillary and mandibular incisors in relation to the facial anterior part (Group A-1) displayed significant density correlation with Group A-2 and high relative connectivity with Groups B and C, likely due to their proximity to anatomical structures. In classical cephalometric analysis, the variables of A-1 are viewed differently, often used as auxiliary measurement variables or considered independently^17–19^, while some studies pair them with groups (A-2, B) that measure the anterior angle^7,8,11–13^. Although A-1 is highly correlated with adjacent groups, it can still be considered an independent anatomical characteristic as there is a practical advantage to studying it separately, based on the network structure.

By applying the MST algorithm to the network structure illustrated in Fig. 3, we can directly grasp the key pairs of variables in the connections between the 10 highly clustered groups of variables, and more information can be obtained by considering the information in Fig. 2a together. The intra-correlations of each cluster exhibit a strong clique or clique-like structure, except for Group D. That is, each of the nine dense clusters independently represents an anatomical feature in the oral and maxillofacial region, which is the main characteristics revealed by various cephalometric analyses.

Group D should not be considered a single anatomical feature because, unlike other groups, it does not have a clique or a cluster structure similar to a clique. Kim’s study introduced the [60] Overbite depth indicator (ODI) which consists of a combination of two variables^14,16,21^, [61] A–B to Mandibular Plane and [14] Palatal Plane angle, and it represents a single anatomical characteristic, the depth of the overbite. However, in the case of [63] combination factor or [64] Extraction index, it is designed as an index that mixes different anatomical characteristics to be used as a basis for decision-making. In Group D, each variable is a subset of the other by definition. Therefore, the strong correlation is limited to the edges of one line and cannot indicate the same anatomical feature. In summary, the analysis of 65 cephalometric measurements showed that nine anatomical characteristics were the primary factors, except for group D as presented in Table 2.

Statistical and advanced network analysis methodologies, weighted network and minimum spanning tree, were employed to visualize the correlation structure among anatomical characteristics in this study. This study process can be standardized and applied as a framework for studying malocclusions of Class I, II, and III as well as different ethnicities. Comparing the outcomes of studies that use identical data sets and analysis framework is expected to improve the value of future research.

## Conclusion

As suggested in this study, using a comprehensive data set, data-based cutoff values, and network analysis tools ensure the homogeneity of the results of the future researches, enabling comparison or integration. Furthermore, data-based analysis was able to uncover important information despite the excessively complex correlations between anatomical characteristics in the oral and maxillofacial region. In some cases, variables representing anatomically identical features derived from different cephalometric analyses could be subdivided. In other cases, variables representing different anatomical features were actually reducible to the same feature. Through all of these analytical processes, 9 groups of variables that could be characterized by identical anatomical features in the oral and maxillofacial region. In conclusion, understanding the correlation structure of the input data set used to profile the anatomical characteristics of malocclusion patients is an essential step in the study of malocclusion using artificial intelligence, and will be valuable for future work.

### Supplementary Information

Supplementary Table S1.

## Data Availability

The data that support the findings of this study are available from Korean Association of Orthodontics, but restrictions apply to the availability of these data, which were used under license for the current study, and so are not publicly available. Data are however available from the authors upon reasonable request and with permission of Korean Association of Orthodontics.
