# Supplementary material for: A study of correlations between cephalometric measurements in Koreans with normal occlusion by network analysis
Source: Sci Rep. 2024 Apr 26;14:9660. doi: 10.1038/s41598-024-60410-1 (PMC11053105; doi:10.1038/s41598-024-60410-1)
Supplement: Supplementary file 1 — Supplementary Table S1. [file 41598_2024_60410_MOESM1_ESM.docx]

Table. S-1 The overall list of lateral cephalometric variables of cephalometric analysis widely known as Ricketts, Downs, Steiner, Wits, Tweed, Jarabak, McNamara and Kim (without overlap)

| **Method** | **Cephalometric variables(unit)** | **Definition** | **Index** ^a^ |
| --- | --- | --- | --- |
| Ricketts | Molar Relation (mm) | The distance between the distal surfaces of the lower and upper molars measured along the occlusal plane. |  |
|  | Canine Relation (mm) | The distance between tips of lower and upper canines measured along occlusal plane. |  |
|  | Incisor Overjet (mm) | The distance between tips of the upper and lower incisors measured along the occlusal plane | 1 |
|  | Incisor Overbite (mm) | The distance between the tips of the lower and upper incisors measured perpendicular to the occlusal plane. | 2 |
|  | Mandibular Incisor Extrusion (mm) | The distance between the tip of the lower incisor and the occlusal plane. |  |
|  | Interincisal Angle (º) | The angle formed by the long axes of the central incisors. | 3 |
|  | Convexity of Point A (mm) | The distance between Point "A" and the facial plane. | 4 |
|  | Lower Facial Height (º) | The angle from anterior nasal spine to the center of the ramus(Xi) to PM | 5 |
|  | Upper Molar to PTV (mm) | The distance from the pterygoid vertical(the back of the maxilla) to the distal of the upper first molar. | 6 |
|  | L1 to A-Pog (mm) | The distance from the tip of the lower incisor to the line defining the jaws, the "A-PO" plane. | 7 |
|  | U1 to A-Pog (mm) | The distance from the tip of the upper incisor to the "A-PO" plane. | 8 |
|  | L1 Inclination (º) | The angle between the long axis of the lower incisor and the "A-PO" plane. | 9 |
|  | U1 Inclination (º) | The angle between the long axis of the upper incisor and the "A-PO" plane. |  |
|  | Occlusal Plane to Ramus (mm) | The distance between the occlusal plane and the center of the ramus(Xi). Positive numbers indicate the occlusal plane is above Xi point, nagative below Xi point. |  |
|  | Occlusal Plane Inclination (º) | The angle between the corpus axis and the occlusal plane(counter clockwise). |  |
|  | Lower Lip to E-Plane (mm) | The distance between the lower lip and the esthetic (nose-chin) plane. | 10 |
|  | Upper Lip Length (mm) | The distance between anerior nasal spine and the embrasure of the lips. |  |
|  | Lip Embrasure - Occlusal Plane (mm) | The distance between the embrasure of the lips and the occlusal plane. Negative values indicate the occlusal plane is below the lip embrasure. |  |
|  | Facial Angle (º) | The angle between the facial plane and Frankfort Plane. Downs facial angle. | 11 |
|  | Facial Axis (º) | The angle between facial axis(Pt-Gn) and cranial axis(Ba-N) | 12 |
|  | Facial Taper (º) | The mandibular plane mesaured to the facial plane. |  |
|  | FMA (º) | The angle between Frankfort Horizontal Plane and the Mandibular plane. | 13 |
|  | Maxillary Depth (º) | The angle formed by the Frankfort Plane and the plane from Nasion to Point A. |  |
|  | Maxillary Height (º) | The angle formed by the points Nasion, CF(the intersection of Frankfort and PTV) and "A point." |  |
|  | Palatal Plane Angle (º) | The angle between Frankfort Plane and the palatal plane. | 14 |
|  | Cranial Deflection (º) | The angle between the Basion-Nasion and Frankfort Planes. |  |
|  | Cranial Length – Anterior (mm) | The distance between CC point and Nasion. |  |
|  | Posterior Facial Height (Ricketts) (mm) | The distance between gonion and CF point. |  |
|  | Ramus Position (º) | The angle between the Frankfort Plane and the CF-Xi plane. |  |
|  | Porion Location (mm) | The distance between Porion and the PTV. |  |
|  | Mandibular Arc (º) | The angle between the corpus and condyle axes. | 15 |
|  | Corpus Length (mm) | The distance between Xi and the intersection of the lines Xi-PM and and A-PO. |  |
| Downs | Facial convexity (º) | The angle between Nasion – A point and A point – Pogonion Line | 16 |
|  | A-B Plane Angle (º) | Point A-Point B to Nasion-Pogonion Angle | 17 |
|  | Y Axis (º) | The angle between Sella Gnathion to Frankfurt Horizontal Plane. | 18 |
|  | Occlusal Plane Angle (º) | The angle of cant of occlusal plane in relation to Frankfurt Hoizontal Plane. | 19 |
|  | L1 to Occlusal plane angle (º) | The angle between line through long axis of Lower Incisor and occlusal plane. | 20 |
|  | L1 to Mandibular plane(90-) (º) | The angle between line through long axis of Lower incisor and Mandibular plane. |  |
| Steiner | SNA (º) | Sella-Nasion to A Point angle. | 21 |
|  | SNB (º) | Sella-Nasion to B Point angle. | 22 |
|  | ANB (º) | A point-Nasion to B Point angle. | 23 |
|  | SND (º) | Sella-Nasion to D Point Angle. |  |
|  | U1 to NA (mm) | The distance from upper incisor to NA line | 24 |
|  | U1 to NA Angle (º) | The angle between upper incisor to NA line | 25 |
|  | L1 to NB (mm) | The distance from lower incisor to NB line | 26 |
|  | L1 to NB Angle (º) | The angle between lower incisor to NB line | 27 |
|  | Pog to NB (mm) | The distance from Pog to NB line | 28 |
|  | Pog & L1 to NB (diff.) | Ratio between lower incisor to NB and Pogonion to NB | 29 |
|  | S to L (mm) | The distance from L point to Sella |  |
|  | S to E (mm) | The distance from E point to Sella |  |
|  | SN-GoGn (º) | The angle of line SN and GoGn |  |
|  | Occlusal Plane to SN (º) | The angle between occlusal plane and SN plane | 30 |
| Wits | Wits Appraisal (mm) | Perpendicular lines dropped from points A and B onto the occlusal plane. Wits reading is measured from AO to BO | 31 |
| Tweed | IMPA (º) | Angle between long axis of lower incisor and mandibular plane angle | 32 |
|  | FMIA (º) | Frankfort mandibular incisor angle | 33 |
| Jarabak | Saddle Angle (º) | The angle between Na-S-Ar | 34 |
|  | Articular Angle (º) | The angle between Sella-Ar-Go | 35 |
|  | Gonion Angle (º) | Angle between Ar-Go-Gn | 36 |
|  | Sum (º) | Saddle Angle + Articular Angle + Gonion Angle | 37 |
|  | Anterior Cranial Base (mm) | The distance from Nasion to Sella | 38 |
|  | Posterior Cranial Base (mm) | The distance from Sella to articulare | 39 |
|  | Upper Gonial Angle (º) | The angle between N-Go-Ar | 40 |
|  | Lower Gonial Angle (º) | Angle between N-Go-Gn | 41 |
|  | Ramus Height (mm) | The distance from Articulare to Gonion | 42 |
|  | Posterior Cranial Base to Ramus ratio | The ratio of ramus height and posterior cranial base | 43 |
|  | Mandibular Body Length (mm) | The distance from Gonion to Menton | 44 |
|  | Body to Anterior Cranial Base Ratio | The ratio of Body length and anterior cranial base | 45 |
|  | Facial Depth (mm) | The distance from Nasion to Gonion | 46 |
|  | Facial Length on Y-Axis (mm) | The distance from Sella to Gnathion | 47 |
|  | Y-Axis to SN (º) | Angle of line SN and SGn |  |
|  | Posterior Facial Height (mm) | The distance from Sella to Gonion | 48 |
|  | Anterior Facial Height (mm) | The distance from Nasion to Menton | 49 |
|  | Posterior to Anterior Facial Height ratio | Ratio of posterior facial height to anterior facial height | 50 |
|  | Facial Plane Angle (º) | The angle of line SN and NPog |  |
|  | Occlusal Plane to GoGn (º) | Occ. Plane-GoGn | 51 |
|  | L1 to GoGn (angle) (º) | The angle of lower incisor and GoGn plane |  |
|  | L1 to GoGn (linear) (mm) | The distance from tip of lower incisor to GoGn plane |  |
|  | U1 to SN Angle (º) | The angle of Maxillary incisor and SN plane | 52 |
|  | U1 to Facial Plane (mm) | The distance from Maxillary insior tip to facial plane | 53 |
|  | L1 to Facial Plane (mm) | The distance from Mandibular insior tip to facial plane | 54 |
|  | Lower Anterior Facial Height (mm) | The distance from ANS to Menton | 55 |
|  | ANS-Me. / Nasion-Me. | The ratio of ANS-Menton and Nasion-Menton |  |
|  | Upper Lip to E-Plane (mm) | The distance from upper lip to E-plane(nasal tip-soft tissue Pogonion) | 56 |
| McNamara | Nasion perpendicular to point A (mm) | The distance from A point to Nasion perpendicular. | 57 |
|  | Mandibular Length (mm) | The distance from Condylion to Gnathion |  |
|  | Midfacial Length (mm) | Distance from Condylion to A point |  |
|  | Maxillomandibular Differential (mm) | Difference between Effective mandibular length and Effective midfacial length. |  |
|  | Pog-N Perpendicular (mm) | The distance from Pogonion to Nasion perpendicular | 58 |
|  | U1 to A Vertical (mm) | The distance from the facial surface of the upper incisor to a vertical line through point A, parallel to the nasion perpendicular. | 59 |
|  | Upper Pharyngeal Width (mm) | The distance from a point on the posterior outline of the soft palate to the closest point on the posterior pharyngeal wall. |  |
|  | Lower Pharyngeal With (mm) | The distance from the intersection of the posterior border of the tongue and the inferior border of the mandible to the closest point on the posterior pharyngeal wall. |  |
| Kim | ODI (º) | A-B to Mand. Plane + Palatal Plane angle | 60 |
|  | A-B to Mandibular Plane (º) | The angle of AB-Mandibular plane | 61 |
|  | APDI (º) | Facial plane to FH plane angle +- A-B plane to facial plane angle * +- palatal plane to FH plane angle ** | 62 |
|  | Combination Factor (º) | ODI + APDI | 63 |
|  | Extraction Index | ODI + APDI - (130-interincisal angle)/5 - (Upper Lip + Lower Lip E-Plane) | 64 |
|  | U1 to FH (º) | The angle between Maxillary central incisor and FH line. | 65 |

^a^ Index means the variable number used in this study.
